# Systemic Lupus Erythematosus in Children and Young People

**DOI:** 10.1007/s11926-021-00985-0

**Published:** 2021-02-10

**Authors:** A. Charras, E. Smith, C.M. Hedrich

**Affiliations:** 1 Department of Women’s & Children’s Health, Institute of Life Course and Medical Sciences, University of Liverpool, Liverpool, UK; 2 Department of Paediatric Rheumatology, Alder Hey Children’s NHS Foundation Trust Hospital, Liverpool, UK; 3 Institute in the Park, Alder Hey Children’s NHS Foundation Trust Hospital, East Prescot Road, Liverpool, L14 5AB UK

**Keywords:** Juvenile onset, Childhood, Systemic lupus erythematosus, Genetics, Epigenetics, Pathophysiology, Treatment, Classification

## Abstract

**Purpose of Review:**

Juvenile-onset systemic lupus erythematosus ((j)SLE) is an autoimmune/inflammatory disease that results in significant damage and disability. When compared to patients with disease onset in adulthood, jSLE patients exhibit increased disease activity, damage and require more aggressive treatments. This manuscript summarises age-specific pathogenic mechanisms and underscores the need for age group–specific research, classification and treatment.

**Recent Findings:**

Genetic factors play a significant role in the pathophysiology of jSLE, as > 7% of patients develop disease as a result of single gene mutations. Remaining patients carry genetic variants that are necessary for disease development, but require additional factors. Increased ‘genetic impact’ likely contributes to earlier disease onset and more severe phenotypes. Epigenetic events have only recently started to be addressed in jSLE, and add to the list of pathogenic mechanisms that may serve as biomarkers and/or treatment targets. To allow meaningful and patient-oriented paediatric research, age-specific classification criteria and treatment targets require to be defined as currently available tools established for adult-onset SLE have limitations in the paediatric cohort.

**Summary:**

Significant progress has been made in understanding the pathophysiology of jSLE. Meaningful laboratory and clinical research can only be performed using age group–specific tools, classification criteria and treatment targets.

## Introduction

Juvenile-onset systemic lupus erythematosus (jSLE) is a rare but severe multisystem autoimmune/inflammatory disease that can affect any organ system and cause significant damage, disability and/or death. It is defined by disease onset before the age of 18 and affects approximately 15–20% of SLE patients [1–5]. The incidences of jSLE ranges between 0.36 and 2.5 per 100,000 children, with a prevalence of 1.89–34.1 per 100,000 [6–10]. Compared to adult-onset SLE, jSLE is more aggressive, with higher disease activity and medication burden (including corticosteroids and other immunosuppressive drugs) that contributes to the increased morbidity and mortality associated with the disease [1, 11, 12], more severe organ manifestations, the presence of greater damage at the time of diagnosis, and a higher incidence of renal, cardiovascular and neuropsychiatric involvement [1, 12–14]. Overall standardised mortality rates are higher in SLE when compared to the general population (SMR 2.2 across all ages), and in patients under the age of 18 in particular, the SMR is approximately three times higher than normal (SMR 6.5) [15]. Diagnosis and treatment of jSLE can be difficult and is complicated by marked heterogeneity between individual jSLE patients in terms of disease presentation and progression, treatment response, and in their overall disease severity with some experiencing mild disease and others having life-threatening manifestations [16].

Here, we will discuss age-related factors contributing to the clinical phenotype and disease progression in jSLE, including genetic and epigenetic alterations; we will summarise new developments in patient stratification and treatment options, and touch on future research directions and initiatives to improve quality of life and outcomes in jSLE.

### Impact of Age on Disease Presentation

The peak age of jSLE onset is 12.6 years [16]. Patients with very early disease onset (before 5 years of age) are more likely to display an atypical presentation (e.g. lack of autoantibodies), more severe disease courses and poor prognosis [2, 15, 17–19]. A recent study involving 418 jSLE patients from the UK showed that, at diagnosis, adolescent jSLE patients (14–18 years) presented with a higher number of American College of Rheumatology (ACR) 1997 classification criteria when compared to pre-pubertal (≤ 7 years) and peri-pubertal patients (8–13 years), with higher levels of mucocutaneous, musculoskeletal, renal and cardiorespiratory disease activity (all *p* < 0.05). Adolescent jSLE patients (> 13 years) also differed from younger age groups (peri-pubertal, 8–13 years; pre-pubertal, < 8 years) in terms of serological disease patterns, displaying more frequent ANA positivity and higher anti-dsDNA titres (both *p* < 0.05). The youngest patient group less frequently presented with leukopenia (*p* = 0.002), thrombocytopenia (*p* = 0.004) and/or low complement (*p* = 0.002) when compared to older age groups. The present study supports the hypothesis that patients diagnosed with jSLE during adolescence may display a more ‘classic’ SLE phenotype due to variation in the pathogenic mechanisms at different ages, accounting for the more atypical SLE presentation seen in younger patients [20].

### Ethnic Differences, Disease Presentation and Course

Studies in adult-onset SLE cohorts have demonstrated that ethnicity has a strong impact on disease course and outcomes [10, 21–28]. A very recent study from the UK has similarly confirmed this to be the case in jSLE, with Black African/Caribbean jSLE patients showing more ‘classical’ laboratory and clinical features when compared to White Caucasian or Asian patients at diagnosis. The study also showed that Black African/Caribbean jSLE patients exhibit more renal involvement and more frequently receive cyclophosphamide and rituximab during their disease course when compared to patients of other ethnicities. Similar to adult studies, jSLE was found to be more prevalent in patients from minority ethnic backgrounds, compared to the UK national census figures for prevalence of ethnic minorities in the population as a whole, with 51% of UK jSLE Cohort Study participants being Caucasian, compared with 86% of the UK population as a whole [29]. Of interest, the present study also showed higher numbers of male patients and less ANA and/or anti-dsDNA positivity among Caucasians (as compared to patients from minority ethnic backgrounds), which may be due to the presence of more ‘atypical’ and/or ‘monogenic’ disease in Caucasians [2, 4, 29].

### The Molecular Pathophysiology of jSLE

Several factors, including familial clusters, aforementioned ethnicity distribution with over-representation with minority ethnic groups, age-specific differences in clinical and laboratory phenotypes when compared to adults, and more severe and difficult-to-treat organ manifestations suggest variable pathogenic mechanisms between age groups, and a stronger involvement of genetic factors in childhood. However, especially as genetically identical monozygotic twins only exhibit disease concordance in 40–60% or all cases, additional factors appear to be involved in the pathophysiology of jSLE, further complicating the situation [2, 4, 30–32].

### Genetic Factors

Genetic mutations or polymorphism, aneuploidy (abnormal number of chromosomes) and copy number variations are events that can cause or contribute to disease, while allowing for phenotypic variation in human population and (at least partially) explain complex inheritance.

#### Gene Mutations and Monogenic Disease

As briefly mentioned above, familial clustering of SLE patients, (relatively) high disease concordance in monozygotic twins (40–60%), and increased risk and poor prognosis of individuals of African or Asian descent suggest that genetic factors play a critical role in the pathogenesis of SLE [5, 33]. However, especially based on the observation that disease penetrance is limited (e.g. concordance rates in genetically identical twins), SLE has been identified as a pathophysiologically highly complex condition in which gene mutations, polymorphisms and additional factors may be involved.

Indeed, only a relatively small number of patients diagnosed with SLE (estimated 1–4% across all age groups) carry highly penetrant mutations in single genes that are strong enough to cause disease. So-called monogenic SLE is caused by mutations in genes involved in the complement pathway, nucleic acid sensing and processing, apoptosis, and/or lymphocyte activation [2].

While the exact molecular pathophysiology is not known for all, mutations affecting the early the *complement pathway* (*C1q, C1r, C1s, C2, C4A* and *C4B*) [34–39] result in inflammation and immune activation through incompletely understood mechanisms. Defective clearance of immune complexes results in their deposition in peripheral tissues, local inflammation, cytokine expression (including type I interferons) and immune cell infiltration which amplifies the above. Another mechanism may be the altered negative selection of self-reactive B lymphocytes in complement C4 deficiency. Indeed, insufficient clearance of cellular debris, which is dependent on complement activation, is a key mechanism not only in primary complement deficiencies but also in more common ‘classical’ forms of SLE [2, 4, 30, 40]. Recently, a genome-wide analysis on a large British-French jSLE cohort investigated rare mono-allelic variants, further highlighting the importance of the complement pathway [41•].

*Disturbed apoptosis* may be involved in SLE and other autoimmune/inflammatory conditions, and mutations in the *FAS* (Fas cell surface death receptor) or *FASL* (Fas ligand) [42, 43] genes, regulators of activation-induced cell death, result in autoimmune lymphoproliferative syndrome (ALPS). Mice deficient of Fas (MRL.*lpr* mice) are prone to SLE-like disease and generalised lymphoproliferation. In both humans with gene mutations and genetically modified mice, ineffective elimination of T lymphocytes results in lupus-like disease, systemic inflammation, and tissue and organ damage [2, 4, 30, 40, 44, 45].

A number of genes affecting *nucleic acid metabolism and sensing* have been linked with *increased type I interferon expression*, the resulting presence of a so-called interferon signature, systemic inflammation and clinical pictures that (more or less) resemble SLE [2, 4, 40]. Impaired processing and removal of chromatin components (including DNA) contribute to autoantibody production and tissue damage (as also happens in aforementioned complement deficiencies). In humans and mice deficient in DNAse1, accumulation of extracellular chromatin contributes to immune activation, type I interferon expression, autoantibody production and subsequently lupus-like disease. Rare familial cases of SLE segregate with autosomal recessive mutations in DNase1 (deoxyribonuclease 1) or DNASE1L3 (deoxyribonuclease 1 like 3*,* a homologue of DNAse1), extracellular accumulation of DNA, autoantibody production, complement consumption and early-onset SLE [44, 45]. Loss-of-function mutations in the gene encoding for the repair exonuclease TREX1 (three prime repair exonuclease 1) result in uncontrolled type I interferon expression and the clinical phenotype described as familial chilblain lupus that is characterised by painful and sometimes ulcerating chilblain lesions. Loss of TREX1 results in cytoplasmic accumulation of single-stranded DNA, which is detected by the nucleic acid sensing machinery resulting in type I interferon release. Thus, *DNASE1*, *DNASE1L3* and *TREX1* mutations are key representatives of primary type I interferonopathies, some of which share clinical characteristics with SLE [2, 4].

All of the aforementioned (and additional) monogenic SLE-like diseases following Mendelian inheritance are usually characterised by *disturbed apoptosis*, with mutations in the *FAS* (Fas cell surface death receptor) or *FASL* (Fas ligand) [42, 43] genes, regulators of activation-induced cell death, resulting in autoimmune lymphoproliferative syndrome (ALPS). Mice deficient of Fas (MRL.*lpr* mice) are prone to SLE-like disease and generalised lymphoproliferation. In both humans with gene mutations and genetically modified mice, ineffective elimination of T lymphocytes results in lupus-like disease, systemic inflammation, and tissue and organ damage [2, 4, 30, 40, 44, 45].

Relatively recently discovered and explored mutations in *PKCD* (protein kinase C delta), which plays a role in cell apoptosis and proliferation, but is also involved in B-cell negative selection, segregate with SLE-like disease likely affecting the same or closely related pathways as the above [46–49].

Taken together, rare gene mutations affecting innate or adaptive immune signalling can result in SLE-like clinical phenotypes. Genetic forms of SLE/SLE-like disease may be over-represented in patients with ‘early-onset SLE’, which is characterised by disease expression within the first years of life [19]. Characteristically, early-onset SLE patients present with severe and sometimes ‘not classical’ symptoms of SLE (such as lack of autoantibodies), and can show poor response to routine treatment [2, 5, 19, 40]. In addition to aforementioned genes, genome-wide association studies (GWAS) and targeted approaches have revealed associations between mutations in one of more than 40 genes and monogenic SLE-like conditions [2, 4].

#### Gene Polymorphisms and Risk Alleles

As mentioned above, only few patients diagnosed with SLE carry disease-causing mutations in single genes. Most individuals have a genetically determined risk for the development of SLE (e.g. disease-associated risk alleles) that requires additional factors to be present or accumulate over time to result in clinical disease [2, 4, 5, 40]. Genome-wide association studies (GWAS) and targeted approaches, conducted mainly in adult-onset SLE cohorts, have revealed associations between polymorphisms in multiple genes, some of which are involved in B- and/or T-cell activation, neutrophil and monocyte biology, TLR (Toll like receptor) and interferon signalling, inflammation, immune complex processing and cell clearance [50]. Some of these variants were assessed and confirmed in jSLE cohorts and associations were identified with some patient-disease characteristics (summarized in Table 1).

**Table 1:** Gene variants associated with jSLE

Gene	Variant	Pathways involved	Description	Ethnicity/origin	References
*C1q*	rs292001	Immune complex clearance	Mutation associated with lupus nephritis development	Egyptian	[51]
*ETS1*	rs4937333	Immune cell signalling	Mutation associated with proteinuria	Japanese	[52]
HLA-DRB1	HLA-DRB1*15 (15 g) allele	B and T cells signalling	Risk factor for SLE	Egyptian	[53]
	HLA-DRB1*17, HLA-DRB1*10, HLA-DRB1*15 and HLA-DRB1*07 alleles		Contribution of HLA- DRB1 alleles related to renal histologic classes, especially class I, class II A, class II B and class V	Brazilian	[54]
*IFIH1*	rs2111485	TLR/IFN signalling	Lupus nephritis-protective effect	Multi-ethnic cohort	[55•]
*IL1β*	rs16944	Immune cell signalling	Risk factor	Iranian	[56]
*IL10*	rs1800871, rs1800872	Immune cell signalling	Increase the susceptibility to nephritis for GCC haplotype	Thai	[57]
*IL1B*	rs1143629	Immune cell signalling	Disease development	Brazilian	[58]
*IL17A*	rs2275913, rs763780, rs2397084	Immune cell signalling	Risk factor	Egyptian	[59]
*IRAK1*	rs10127175	TLR/IFN signalling	Risk factor	Multi-ethnic cohort	[60]
	rs2239673,rs763737, rs5945174 and rs7061789		4 SNP haplotypes (GGGG) being strongly associated with the disease in 3 (African Americans, Asian Americans and Hispanic Americans) of 4 different ethnic groups (not on European American)	Multi-ethnic cohort	[61]
*IRF5*	rs2004640, rs10954213, rs2004640, rs2280714	TLR/IFN signalling	rs2004640 increases risk of nephritis development	Egyptian	[62]
	rs729302, rs11768806, rs4728142,rs3807135, rs2004640, rs752637,rs3807306, rs2280714		Risk factor	Multi-ethnic cohort	[63]
*JAZ1F*	rs10245867		Risk factor	Multi-ethnic cohort	[55•]
*MBL2*	rs7096206	Complement pathway	Higher risk of cutaneous manifestations and pleuritis/pericarditis	Hungarian	[64]
*NRF2*	653G/A	Oxidative stress	Nephritis in childhood onset female SLE patients	Mexican	[65]
*PTPN22*	rs2476601	Immune cell signalling	Risk factor	Mexican	[66]
	rs1217407		Risk factor	Multi-ethnic cohort	[67]
*SELP*	rs3917815	Immune cell signalling	Risk factor	Multi-ethnic cohort	[60]
*SSP1*	rs9138	TLR signalling	Association with proteinuria	Japanese	[52]
*STAT1*	c862A > G; p.T288A	Immune cell signalling	Risk factor	–	[68]
*STAT4*	rs7582694	Immune cell signalling	Disease manifestation (malar rash, photosensitivity, hair falling, increase 24 h protein in urine, ANA+, dsDNA and anti-Sm detection and decreased of C3 and C4) and higher SLEAI and damage index	Egyptian	[69]
	rs7574865		Association with malar rash	Japanese	[52]
*TNFAIP3*	rs2230926	NF-κB signalling	Associated with SLE in male subgroup	Japanese	[52]
*TNF*	308-A	immune cell signalling	Risk factor	Mexican	[70]
	863C > A		Nephritis and Raynaud phenomenon	Iranian	[71]
UBE2L3	rs131658	NF-κB signalling	Association with lupus nephritis	Multi-ethnic cohort	[55•]

#### Ancestry-Specific Genetic Polymorphisms and Disease Susceptibility

As mentioned above, SLE patients of different ethnic backgrounds exhibit significant differences in clinical disease presentation, treatment response and disease course [2, 4, 5]. These clinical observations are underpinned by several known genetic associations.

In contrast to Caucasian and Asian populations, the rs2304256, rs280500 and rs12720270 variants in *TYK2* are not associated with jSLE in Mexican populations. Moreover, rs12720356 and rs34536443 variants have a lower frequency in Mestizos as compared to ‘Spaniards’ and are absent or rare in indigenous populations, suggesting that the presence of these alleles in the entire Mexican population was introduced by Spaniards. Thus, the authors claim that Mexican Mestizos may have inherited higher frequencies of SLE risk alleles from the indigenous population, while protective variants may have been subject to negative selection [72]. In the same *Mestizo* (person of combined European and Indigenous American descent) population, Ramirez-Bello et al. did not observe associations between *FCRL3* (Fc receptor like 3) variants and jSLE, while associations exist in European and some Asian populations [73]. In a recent paper, Webber et al. described an association between several SLE risk alleles and lupus nephritis risk in children with a European background [55•].

A better understanding of the contribution of ethnicity-related genetic risk, clinical presentations and associated outcomes will improve the understanding of disease pathophysiology, allow for patient stratification and individualised treatment, as well as outcome assessment.

#### The Contribution of Risk Alleles to Early Disease Onset in SLE

As children and young people with jSLE, in the absence (or at least with fewer) of comorbidities and environmental impacts accumulated, exhibit more severe clinical phenotypes and reduced response to standard treatment when compared to adult-onset SLE patients, increased genetic risk is likely contributing. Approximately 7–8% of children [41•] (unpublished data from the UK jSLE cohort study) exhibit monogenic disease that classifies as SLE. This percentage is higher as compared to the overall SLE population across age groups (estimated 1–4%), but only explains a relatively small fraction of cases [2, 5]. Thus, an increased number of risk alleles have been proposed contributing to jSLE.

Webb et al. reported an increased number of SLE-associated polymorphisms in jSLE patients when compared to Gullah and African-American adult-onset SLE patients. The authors therefore concluded that genetic risk has a key role in determining age of disease onset in SLE patients with SLE of African descent, which is also an important predictor of disease severity [74]. Similarly, Joo et al. calculated Genetic Risk Scores (GRS) in a Korean cohort and demonstrated that jSLE is associated with a higher GRS when compared to adult-onset SLE [75]. Lastly, in a recent study, Webber et al. observed that effect of both non-HLA and HLA GRS for the development of lupus nephritis were higher in patients with juvenile- as compared to adult-onset SLE. However, differences did not reach statistical significance [55•]. Moreover, some variants were distinct between juvenile- and adult-onset SLE (Table 2).

**Table 2:** Genetic variants associated explicitly with juvenile vs. adult-onset SLE

Gene	Variant	Pathway involved	Description	Ethnicity	References
*ESR1* and *ESR2*	rs2234693, rs4986938	Oestrogen-related pathways	Two distinct associations, an association between ESR1 polymorphism and jSLE, and between ESR2 and aSLE	Polish	[76]
*ORα*	Polymorphism	Oestrogen-related pathways	Association with age at disease onset	Korean	[77]
*MBL2*	rs7096206	Complement pathways	Could be strongly associated with juvenile onset of SLE and also related to specific organ involvement	Hungarian	[64]
*STAT4*	rs7574865, rs7601754	Immune cell signalling	Lack of association with susceptibility to JSLE in Iranian population, despite their association with the risk of adult SLE in the same population	Iranian	[78]
*MECP2*	rs1734787, rs1734791	Chromatin regulation	SLE susceptibility variants in Iranian population. However, none of them was associated with JSLE risk	Iranian	[79]
*PDCD1*	PD1.3A	T-cell signalling, NF-κB signalling, adaptive immune system	Weaker association of this SNP with childhood-onset SLE female patients compared with that reported by Prokunina et al, in Mexican female adults with SLE	Mexican	[80]

#### Aneuploidy as a Genetic Risk Factor for SLE

Aneuploidy is defined as an abnormal number of (entire or parts of) chromosomes in a cell, tissue or entire organism due to abnormal meiosis [81].

The X chromosome contains a number of genes involved in the regulation of innate and adaptive immune responses, including *TLR7*, *TLR8*, *IRAK1*, *IL2RG*, *FOXP3* and *CD40L*. Studies targeting sex-related differences of immune responses investigated effects mediated by the number of X chromosomes and delivered an increased risk for the development of SLE with growing numbers of X chromosomes.

In males (physiologically having one X and one Y chromosome), the presence of an additional X chromosome, such as 46, XX in la Chapelle’s syndrome or 47, XXY in Klinefelter’s syndrome, is associated with an increased risk of SLE. This risk is similar to euploid women (46, XX); and no differences in SLE disease phenotypes between aneuploid men with an additional X chromosome and euploid women were observed [82, 83]. The prevalence of SLE in males with Klinefelter’s syndrome is nearly 14-fold higher when compared to boys/men with 46, XY karyotypes [84]. In 2016, Liu et al. reported an increased prevalence (~ 2.5 times higher than in women 46, XX and ~ 25 times higher than in men 46, XY) of SLE in a cohort of females with an additional X chromosome (47, XXX karyotype) [85]. Conversely, the prevalence of SLE in females with Turner’s syndrome (45, X0 karyotype) is lower when compared to women with 46, XX karyotypes [83]. Recently, Webb et al. reported that the presence of two X chromosomes, independent of serum sex hormones, may be responsible for increased production of type 1 interferons by plasmacytoid dendritic cell as a result of TLR7 stimulation, which may centrally contribute to the increased prevalence of SLE in females [86]. However, additional laboratory investigation is needed to sufficiently understand the involvement of X chromosomes and X chromosome gene dose effects in SLE.

In addition to aneuploidy of the X chromosome, also aneuploidy and mosaicism of chromosome 9 has been reported in SLE patients. Zuang et al. described a familial cluster of SLE patients with a chromosomal translocation involving chromosome 9. The authors concluded that patients’ autoimmune phenomena relate to having three copies of the type 1 *IFN (Interferon)* cluster located on the p (short) arm of chromosome 9, as they also observed increased IFN-α/β and IFN receptor signalling in patients [87]. A mosaic tetrasomy affecting a 42-Mb spanning region on chromosome 9p24.3q12 was observed in a 6-year-old girl with myositis and lupus-like features. This 42-Mb region includes 495 genes, among them 26 encoding for interferon (IFN) pathway related genes [88]. Overall, these reports support the hypothesis that abnormal regulation of type I IFN production is involved in the pathogenesis of SLE, especially in children.

Increased DNA damage and genomic instability are possible outcomes of chromosome gain that can trigger inflammation and result in SLE-like phenotypes. The exact underlying mechanisms, however, remain to be addressed in future studies [81].

#### Copy Number Variation

Copy number variation (CNV) is caused by the loss or gain of genomic segments. Classically, CNVs are defined as events that affect genomic regions longer than 1 kb. It can be observed in healthy individuals, but has ‘gene dose’ effects affecting susceptibility and outcomes in autoimmune disease and beyond. CNV is common across healthy populations with allelic properties similar to aforementioned SNPs (single-nucleotide polymorphisms). A recent study highlights that both rare and common CNVs can have a biological impact in health and disease [89].

Low total *C4*, *C4A* and *C4B* gene copy numbers are associated with an increased risk for the development of jSLE and associated pericarditis (low total *C4*, *C4A*) [90].

In Mexican populations, increased copy numbers of *TLR7* are a susceptibility factor for jSLE, which especially affects male patients, providing additional evidence for the role of X-linked gene dose effects in SLE [91].

As highlighted by Bueno Barbosa et al. in their study on adult-onset SLE, evaluation of the fine-scale architecture of CNV regions, as well as the prediction of pathogenicity of long segments encompassing several homozygous variants found, will contribute to understanding how risk loci harbouring CNV segments affect the aetiology and pathology of SLE [92].

Altogether, the identification of single-nucleotide polymorphism as well as larger extended haplotypes that may include aneuploidy and/or copy number variation will result in a better understanding of pathomechanisms in SLE and resulting disease phenotypes.

### Epigenetic Factors

Genetic variation affects the risk for SLE across ages. However, with the exception of rare monogenic SLE-like conditions, gene variants associated with SLE are not strong enough to confer disease, and additional factors must be accumulated over time to cause disease in a genetically predisposed individual [2, 5, 32, 40]. Epigenetic mechanisms impact upon DNA accessibility and gene transcription without affecting the underlying gene sequence itself. Three main groups of epigenetic mechanisms are currently investigated including DNA methylation, posttranslational histones modifications and non-coding RNAs. Together (and/or individually), these mechanisms regulate DNA compaction and accessibility. Dysregulation of epigenetic events has been linked with a host of health conditions, including cancer and autoimmune disease. However, data from paediatric patient cohorts (including jSLE) are limited.

#### DNA Methylation

DNA methylation is a stable, heritable but also reversible epigenetic mark. During de novo methylation or after cell division, DNA methylation is mediated through the covalent transfer of a methyl group to the fifth carbon position of the cytosine pyrimidine ring by DNA methyl-transferases (DNMTs). Usually, DNA methylation happens at CpG dinucleotides. DNA methylation is involved in cell differentiation, transposable element silencing and imprinting of genes. Its dysregulation has been linked with carcinogenesis, autoimmune/inflammatory disease and other diseases [93, 94].

In adult-onset JSLE, hypomethylation of *CD70* (encoding for TNFSF7-tumour necrosis factor ligand superfamily member 7) in CD4+ T cells results in increased gene expression and subsequently enhanced B-cell stimulation that contributes to the pathogenesis of SLE [95–98]. Findings may be specific to CD4+ T cells, as Keshavarz-Fathi et al. did not observe statistically significant differences in CD70 promoter methylation of peripheral blood mononuclear cells (PBMCs) from patients with jSLE [99].

Long interspersed nuclear element-1 (LINE-1) are repetitive DNA elements that represent about 21% of the human genome and is often used as a marker of global DNA methylation [100]. In LINE-1 DNA methylation in PBMCs from jSLE patients, Huang et al. observed a significant correlation between disease activity and DNA hypomethylation, mainly in patients with mild to severe disease activity (based on SLEDAI (Systemic Lupus Erythematosus Disease Activity Index)-2000) indicating that hypomethylation may reflect disease. Furthermore, LINE-1 methylation levels were lower in jSLE than SLE and negatively correlated to homocysteine concentration, which is higher in this patient group. Their findings support the idea that disruption of one-carbon unit metabolism and hypomethylation of LINE-1 occur in jSLE [101].

Unfortunately, data on molecular events mediating alterations in jSLE are limited and does (to our knowledge) not include studies investigating global DNA methylation. However, Hofmann et al. [102•] determined increased expression of the transcription factor cAMP response element modulator α (CREMα) in CD4+ T cells from patients with jSLE. This is in line with observations in adult-onset disease, where CREMα has been established as a key driver of epigenetic dysregulation (including DNA methylation) through its interactions with DNMTs, among others, resulting in silencing of the *IL2* gene [31, 103–106], a hallmark of effector T cells in SLE.

#### Posttranslational Histone Modifications

Histones are small, arginine- and lysine-rich proteins, organised in octamers that build complexes with segments of 147 base pairs of genomic DNA that are then referred to as nucleosomes. The N-terminal part of histones (‘tail’) is accessible to posttranslational modifications (methylation of arginine or lysine residues, acetylation, ubiquitylation and SUMOylation of lysine and phosphorylation of serine or threonine groups), which modulate DNA compaction and accessibility. They act sequentially or in combination and define the ‘histone code’ [32, 98, 107]. This code can be read and interpreted by other different families of enzymes, including lysine acetyltransferases, HDACs (histones deacetylases), lysine methyltransferases and lysine demethylases, sensitive to these changes, capable of linking chromatin and reshape its organisation, thus regulating processes such as transcription, DNA replication and repair [108]. Alterations to the histone code are involved in the pathophysiology of autoimmune/inflammatory disorders [32, 98, 107].

In PBMCs from patients with jSLE, mRNA expression of IRF5, IFN-α and Sp1 is increased. Exposure of cells to HDAC (histone deacetylase) inhibitor TSA (trichostatin A) or forced histone acetylase p300 expression repressed *IRF5* promoter activity, suggesting the use of HDACi (HDAC inhibitor) as a potential future therapeutic option in SLE [109].

As mentioned above, recently, Hofmann et al. linked dysregulation of the CREMα (cAMP response element modulator α)/DUSP4 (dual specificity protein phosphatase (DUSP) 4) axis in CD4+ T cells from jSLE patients with effector cytokine expression [102•]. As in adult-onset SLE, the transcription factor CREMα is expressed at increased levels in CD4+ T cells from jSLE patients as compared to matched controls [102•, 103, 106]. In CD4+ T cells from jSLE patients, CREMα induces DUSP4 (dual specificity protein phosphatase 4A) expression through co-recruitment of the transcriptional coactivator p300 that mediates histone acetylation. Increased histone acetylation at *DUSP4* promotes gene expression, subsequently resulting in reduced phosphorylation of the transcription factor STAT5 which in turn mediates increased IL-17A and limited IL-2 expression, a hallmark of SLE-associated effector T-cell phenotypes [102•]. These observations are in line with a number of studies in adult-onset SLE patients linking CREMα overexpression with altered epigenetic marks (DNA methylation and histone modifications) that affect effector cytokine expression [31, 32, 103, 105, 106, 110–112].

#### Non-coding RNAs

Micro-RNAs (miRNAs) are the most widely studied non-coding RNAs. They are small RNA molecules that contain 18–25 nucleotides and control mRNA stability and integrity, thereby fine-tuning between 30 and 80% of human genes [113]. Altered miRNA expression play crucial roles in a variety of pathological processes [114], including autoimmune/inflammatory disease.

Preliminary evidence also links miRNAs with the pathophysiology of jSLE. Lashine et al identified reduced expression of mir-155 in PBMCs from jSLE patients. MiR-155 is involved in PP2AC (protein phosphatase(PP)2Ac) expression, a regulator of IL-2 (interleukin-2) release that has been implicated in the pathophysiology of SLE [115–120]. Downregulation of miR-155 is inversely correlated with SLEDAI scores and proteinuria, and positively correlated with blood leukocyte counts [121]. Thus, delivery of miR-155 may be a potential future therapeutic intervention in SLE to rescue IL-2 expression.

Taken together, epigenetic alterations can be acquired through exposure to the environment. Altered epigenetic marks can contribute to disease expression in individuals genetically predisposed to disease development. Understanding the exact causes and molecular effects of epigenetic alterations will aid in biomarker identification and the prevention and/or treatment of jSLE.

### Treatment, Targets and Trials

#### What Is New in Treatment of jSLE?

Due in large to jSLE being a relatively rare disease, treatment paradigms are often extrapolated from adult SLE. Sufficiently powered randomised controlled trials (RCTs) in jSLE are scarce. Most available treatment options are not targeted, and can cause significant adverse events and toxicity [2, 4], particularly in vulnerable age groups such as children and young people.

Although ‘new’ biologic therapies are used for many autoimmune conditions, there have been several notable setbacks in SLE [9], with only belimumab, a monoclonal antibody targeting the B lymphocyte stimulator (BLYS), licensed for SLE (across ages) in over 50 years [10, 11]. It has been approved for use in active adult-onset SLE patients who display serological activity (elevated anti-dsDNA titres and/or low complement levels), in light of post hoc analysis from the BLISS phase II/III trials, which showed a better response in this patient group [122, 123]. Observational studies have not demonstrated the same difference in efficacy in patients who are serologically active [124, 125].

Very recently, the first trial of intravenous belimumab in active jSLE, the PLUTO study, assessed intravenous belimumab (10 mg/kg), plus standard jSLE therapy versus placebo in 93 jSLE patients. At week 52, a numerically higher proportion of patients receiving belimumab met the primary endpoint of SLE Responder Index 4 (SRI4, 52.8% vs. 43.6%; OR 1.49 (95% CI 0.64 to 3.46)); however, the CI crossed 1 [126]. SRI4 is the primary outcome measure that was used in the original adult-onset SLE Belimumab trial.

The major secondary endpoint was the proportion of patients meeting the Paediatric Rheumatology International Trials Organisation/American College of Rheumatology (PRINTO/ACR) cSLE criteria for response to therapy [127]. These criteria consider the percentage change in five core components, including [1] the physician global assessment, [2] Parent Global Assessment of patient well-being, (3) the SLEDAI score, [4] the Paediatric Quality of Life inventory (PedsQL; physical-functioning domain) and [5] proteinuria. Improvement in the PRINTO/ACR jSLE criteria was measured in terms of an ACR 30 or 50 response [127]. Further major secondary endpoints included the proportion of patients with a sustained SRI4 response and parent-global assessment scores.

A significantly higher proportion of belimumab-treated patients achieved both the PRINTO/ACR 30 (52.8% vs. 27.5%; OR 2.92 (95% CI 1.19 to 7.17)) and PRINTO/ACR 50 (60.4% vs. 35.0%; OR 2.74 (95% CI 1.15 to 6.54)) responses. A sustained SRI4 response was not achieved, but there was a significant improvement in the parent-global score [126]. The present study raises important questions about the applicability of adult SLE outcome measures in jSLE, given the known differences in disease activity, severity and damage demonstrated between paediatric, adolescent and adult SLE [11, 12, 128].

In adult SLE, a phase III trial of subcutaneous belimumab versus placebo (in addition to standard SLE therapy) has been completed, meeting its primary endpoint and demonstrating that in hypocomplementemic, anti-dsDNA-positive SLE patients, weekly SC belimumab significantly improved SRI4 response, decreased severe flare incidence, and reduced corticosteroid use [129]. A paediatric specific study is required, as are further properly powered RCTs of other biologics that are under investigation in adult SLE (e.g. baricitinib, anifrolumab).

#### ‘First do no harm’—Increasing Evidence for the Need to Steroid Spare in jSLE

Glucocorticoids remain the cornerstone of treatment in patients with active SLE and are commonly used for prolonged periods in both jSLE and adult-onset SLE, with highly variable regimens used between centres/clinicians [130]. Patients with jSLE (as opposed to adult-onset SLE) are at increased risk of steroid-related damage. In the US Lupus Outcomes Study, a longitudinal cohort of adults with confirmed SLE, jSLE patients were more likely to report steroid-related damage (OR 1.7, 95% CI 1.1–2.8) in the adjusted analysis as compared to those with adult-onset SLE [131]. As discussed above, biologic trials in SLE have by and large been disappointing as compared to other autoimmune diseases, potentially related to the complex and heterogeneous nature of SLE, and the influence of genetic, environmental and hormonal factors. Consideration of alternative endpoints for SLE clinical trials is increasingly receiving attention, with a recent meta-analysis of the steroid-sparing effects of biological treatments used in placebo-controlled, phase III RCTs, showing that most biological therapies (belimumab, tabalumab and epratuzumab) had a steroid-sparing effect, compared to placebo (pooled RR 1.36 (1.19, 1.56), leading to the suggestion that steroid dose could be included in a composite primary endpoint for SLE clinical trials [132]. Leading SLE experts have argued that problems with existing disease activity measures and treatment response outcomes may partly explain why so many trials have failed to meet their primary outcome, and have advocated for steroid reduction as a pragmatic primary outcome measure, indirectly reflecting improved disease control. A suggestion would be that the minimal clinically meaningful difference in response could become a percentage of steroid reduction (e.g. 50%, as compared to placebo) provided that the steroid reduction was sustained for a clinically relevant time period (e.g. for 6 months) [133]. The suggested doses of steroids in adult SLE treatment recommendations (intravenous, oral and tapering regimens) included in both the 2018 British Society of Rheumatology (BSR) and 2019 EULAR recommendations are lower than those advocated within some jSLE treatment regimens [134]. A specific focus on steroid sparing and monitoring of glucocorticoid toxicity in jSLE is therefore warranted.

#### Future Directions in the Management of jSLE and Rationale for a Treat-to-Target Approach

Treatment in jSLE must aim to prevent permanent organ damage, optimise health-related quality of life (HRQOL) and ultimately improve survival through controlling disease activity, and minimising comorbidities and drug toxicity. ‘Treat to target’ (T2T), in which treatment is escalated or modified in pursuit of a pre-defined target, is part of routine clinical care in many areas of medicine (e.g. rheumatoid arthritis, hypertension, diabetes) [135]. In jSLE, a future T2T clinical trial has been advocated as an opportunity to substantially reform the clinical management of jSLE patients, using existing treatments in a more consistent and structured way, with the aim of aggressively controlling inflammation at an early stage. The TARGET LUPUS research programme (‘*T*argeting disease, *A*greeing *R*ecommendations and reducing *G*lucocorticoids through *E*ffective *T*reatment in *LUPUS*’) has been established in order to develop a future JSLE T2T clinical trial [136, 137]. Steroid sparing and monitoring of glucocorticoid toxicity are key elements of the programme. To this end, it is important to define appropriate treatment targets and outcome measures specifically for use in jSLE, organ domain–driven therapeutic algorithms to be used to achieve the target and the most appropriate study design given the rarity of jSLE. A glucocorticoid toxicity index (GTI) has been developed to assess glucocorticoid-related morbidity and glucocorticoid-sparing ability of other therapies in adults with SLE [138], and a paediatric version of the GTI is eagerly awaited and should be validated in the context of jSLE.

#### Performance of classification criteria for SLE in jSLE

Classification criteria have been primarily developed in adult SLE populations, with the aim of defining a relatively homogenous patient population for inclusion in clinical trials. Initial criteria were developed by The American College of Rheumatology in 1982 [139] and updated in 1997 (ACR-1997) [140]. There were concerns that the ACR criteria may miss some SLE patients, in particular those with lupus nephritis and autoantibody positivity but limited other systemic involvement. Therefore, the Systemic Lupus International Collaborating Clinics (SLICC-2012) group established criteria including 11 clinical criteria and 6 immunological criteria. They also agreed that patients with lupus nephritis and ANA or dsDNA positivity could be defined as SLE, in the absence of other clinical criteria [141]. Studies examining the performance of the SLICC criteria in international adult and jSLE cohorts have shown higher sensitivity, but lower specificity when using the SLICC versus 1997 ACR classification criteria [142–144].

The ACR and European League Against Rheumatism (EULAR) have recently collaborated to develop new classification criteria for SLE (the EULAR/ACR-2019 criteria), with validation performed in adult-onset SLE cohorts [145]. These criteria include ANA positivity as an entry criterion (ANA titre of at least 1:80 on human epithelial type 2 cells or equivalent positive test result) and use a weighted scoring system. Patients must achieve at least 10 points to be classified as having SLE. The EULAR/ACR-2019 criteria show better sensitivity and specificity compared to earlier criteria in adults with SLE [145]. Two studies have assessed the performance of the EULAR/ACR-2019 criteria in jSLE. The first study included 122 jSLE patients and 89 controls (ANA positive patients with other defined rheumatic diseases), and compared ACR-1997, SLICC-2012 and the EULAR/ACR-2019 criteria for SLE. Using a EULAR/ACR-2019 criteria cut-off score of ≥ 10, the new criteria performed less well for specificity at first visit (67.4%) as compared to both the ACR-1997 (83.2%) and SLICC-2012 criteria (80.9%). For sensitivity, the new EULAR/ACR-2019 criteria scored better than ACR 1997 (87.7% vs. 70.5%) and worse than the SLICC criteria (89.3%). An alternative cut-off point for the new EULAR/ACR-2019 criteria was proposed for use in jSLE, with a score of ≥ 13 resulting in higher specificity, positive predictive value and cut-off point accuracy [146].

The second study included 112 SLE patients aged 2–21 years (jSLE and adult SLE) and 105 controls aged 1–19 years (juvenile dermatomyositis, juvenile scleroderma or juvenile systemic sclerosis). The rheumatologist’s diagnosis of SLE served as the gold-standard criterion for identifying SLE patients. They showed the EULAR/ACR-2019 criteria to have significantly higher sensitivity (85% vs. 72%; *p* = 0.023) and similar specificity (83% vs. 87%; *p* = 0.456) than the 1997-ACR criteria. The absolute EULAR/ACR-2019 classification summary scores were higher in non-White than White cases. Sub-analysis showed that the sensitivity of the EULAR/ACR-2019 criteria was not influenced by patient ethnicity, age or gender, whereas the sensitivity of the ACR-1997 criteria was significantly higher in non-White versus White cases [147]. Further studies are warranted to assess the performance of the EULAR/ACR-2019 criteria in children, in particular as younger children with jSLE have been shown to display less ANA positivity [20].

As limited sensitivity and high specificity are wanted to homogenise patient cohorts for clinical trials, new criteria are considered a success, at least for adult-onset SLE. However, provided limited sensitivity and specificity of adult-centric criteria, it remains to be discussed whether paediatric criteria may be needed. Furthermore, it remains to be stressed that classification criteria will miss jSLE patients when used to diagnose disease (which by definition should not happen).

## Conclusions

While we still do not completely understand the molecular pathophysiology of jSLE, major progress has been made over recent years. While only 1–3% of SLE patients across age groups experience disease caused by single gene mutations (monogenic SLE-like disease), this number is significantly higher in children (7–8%). In jSLE patients with ‘classic’ multifactorial disease, increased genetic burden when compared to patients with adult-onset disease contributes to early disease expression and more severe phenotypes. Lastly, epigenetic events that can be the result of environmental exposure contribute to the molecular pathophysiology and (likely) clinical phenotypes. A complete understanding of aforementioned (and potentially ‘new’) pathomechanisms will improve our understanding, and allow the development of biomarkers and individualised treatment options. Indeed, treatment is mostly empirical and based on studies in adult-onset SLE cohorts. Paediatric-specific studies are only emerging, but are key for the improvement of patient care and disease outcomes. For this, treatment targets and inclusion criteria have to be specific for children and young people and require to presence of paediatric rheumatologists in expert groups assembled e.g. by ACR and EULAR, but also industry.

## Abbreviations

ACR: American College of Rheumatology
ALPS: Autoimmune lymphoproliferative syndrome
ANA: Anti-nuclear antibodies
anti-dsDNA: Anti-double-stranded DNA antibodies
BLYS: B LYmphocyte Stimulator
CD70: TNFSF7-tumour necrosis factor ligand superfamily member 7
CNV: Copy number variation
CREMα: cAMP response element modulator α
DNase1: Deoxyribonuclease 1
DNASE1L3: Deoxyribonuclease 1 like 3
DNMTs: DNA methyl-transferases
DUSP4: Dual specificity protein phosphatase 4
EULAR: European League Against Rheumatism
FAS: Fas cell surface death receptor
FASL: Fas ligand
FCRL3: Fc receptor like 3
GRS: Genetic Risk Score
GTI: Glucocorticoid Toxicity Index
GWAS: Genome-wide association studies
HDACi: HDAC inhibitor
HDACs: Histone deacetylases
HRQOL: Health-related quality of life
IFN: Interferon
IL-2: Interleukin-2
jSLE: Juvenile-onset systemic lupus erythematosus
LINE-1: Long interspersed nuclear element-1
miRNAs: Micro-RNAs
PBMC: Peripheral blood mononuclear cell
PedsQL: Paediatric Quality of Life inventory
PKCD: Protein kinase C delta
PP2AC: Protein phosphatase(PP)2Ac
PRINTO/ACR: Paediatric Rheumatology International Trials Organisation/American College of Rheumatology
RCT: Randomised controlled trial
RR: Relative risk
SLEDAI: Systemic Lupus Erythematosus Disease Activity Index
SLICC: Systemic Lupus International Collaborating Clinics
SMR: Standardized mortality rates
SNPs: Single-nucleotide polymorphism
SRI4: SLE responder index 4
T2T: Treat to target
TARGET LUPUS: Targeting disease, Agreeing Recommendations and reducing Glucocorticoids through Effective Treatment in LUPUS
TLR: Toll-like receptor
TREX1: Three prime repair exonuclease 1
TSA: Trichostatin A
